# The Acute Physiological Responses to Traditional vs. Practical Blood Flow Restriction Resistance Exercise in Untrained Men and Women

**DOI:** 10.3389/fphys.2020.577224

**Published:** 2020-09-29

**Authors:** Eduardo D. S. Freitas, Bianca R. A. Galletti, Karolina J. Koziol, Ryan M. Miller, Aaron D. Heishman, Christopher D. Black, Debra Bemben, Michael G. Bemben

**Affiliations:** ^1^Neuromuscular Laboratory, Department of Health and Exercise Science, The University of Oklahoma, Norman, OK, United States; ^2^Sensory and Muscle Function Laboratory, Department of Health and Exercise Science, The University of Oklahoma, Norman, OK, United States; ^3^Bone Density Research Laboratory, Department of Health and Exercise Science, The University of Oklahoma, Norman, OK, United States

**Keywords:** strength exercise, KAATSU, occlusion training, electromyography, lactate, muscle swelling, hematocrit, plasma volume changes

## Abstract

This study compared the acute physiological responses of traditional and practical blood flow restriction resistance exercise (tBFR and pBFR, respectively) and high- and low-load resistance exercise without BFR (HL and LL, respectively), as well as the potential sex differences within the aforementioned exercise methods. Fourteen men and fifteen women randomly completed the following experimental conditions: (1) tBFR, consisting of four sets of 30-15-15-15 repetitions of the bilateral horizontal leg press and knee extension exercises, at 30% of one-repetition maximum (1-RM), with a 13.5 cm wide pneumatic cuff placed at the most proximal portion of each thigh and inflated to a pressure equivalent to 50% of the participant’s total occlusion pressure; (2) pBFR, which was the same as the tBFR condition, except that an elastic band wrapped around the proximal portion of each thigh at a tightness of 7 on a 0 to 10 perceived pressure scale was used to reduce blood flow; (3) LL, same as the tBFR and pBFR protocols, except that no BFR was applied; and (4) HL, consisting of 3 sets of 10 repetitions at 80% of 1-RM, with the same 1-min rest interval between sets and a 3-min rest period between exercises. At baseline, immediately post-, 5 min post-, and 15 min post-exercise, whole-blood lactate (WBL), indices of muscle swelling (muscle thickness and thigh circumference), hematocrit and plasma volume changes, were measured as well as superficial electromyography (sEMG) amplitude during exercise. There were no significant (*p* > 0.05) differences between the tBFR and pBFR exercise protocols for any of the physiological parameters assessed. However, significantly greater (*p* < 0.05) WBL and sEMG values were observed for HL compared to the remaining exercise conditions. Finally, males displayed greater WBL levels than females at 15 min post-exercise. Interestingly, males also displayed significantly (*p* < 0.05) greater sEMG amplitude than females within the low-load trials during leg press, but no significant (*p* < 0.05) sex differences were observed during knee extension. In conclusion, tBFR and pBFR seemed to be capable of inducing the same acute physiological responses. Furthermore, males displayed greater responses than females for some of the physiological parameters measured.

## Introduction

Low-load resistance training combined with blood flow restriction (BFR) has been shown to result in similar neuromuscular adaptations as traditional high-load resistance training without BFR (Karabulut et al., 2010; Laurentino et al., 2012; Kim et al., 2017). Although the precise mechanisms responsible for these positive adaptations remain unclear, several factors such as increased muscle activation (Fatela et al., 2016), metabolic stress (Suga et al., 2010), muscle swelling (Loenneke et al., 2012b), and influencing biomolecular pathways responsible for muscle anabolism (mTOR) and catabolism (myostatin) (Takarada et al., 2000a; Fry et al., 2010; Laurentino et al., 2012; Nakajima et al., 2016) have been proposed. Of note, BFR resistance training elicits positive adaptations while incorporating resistance training intensities as low as 20–30% of an individual’s one-repetition maximum (1-RM), which may offer a potential training strategy to maintain or improve muscular fitness for those unable to train at traditionally recommended intensities above 60% of 1-RM (ACSM, 2011).

When performing BFR resistance exercise, blood flow to the working muscles is reduced by placing either pneumatic cuffs or elastic wraps at the most proximal portion of the exercising limbs (Kim et al., 2012; Wilson et al., 2013; Lowery et al., 2014; Fatela et al., 2018; Patterson et al., 2019). The evidence demonstrating the effectiveness of BFR resistance exercise is rapidly accumulating (Hwang and Willoughby, 2017; Centner et al., 2018), which has led more recent investigations to focus on the development of the most effective BFR resistance exercise protocol. Previous research has examined the influence of cuff width (Ipavec et al., 2018), cuff material (Buckner et al., 2017), and different restrictive pressures (Fatela et al., 2016); however, one question that remains unanswered is whether the type of restrictive device used to reduce blood flow results in distinct acute physiological responses, potentially manifesting into different long-term adaptations. For example, laboratory and traditional settings commonly use the KAATSU or Hokanson electronic devices, which allow practitioners to precisely control the restrictive pressure applied during exercise. However, the access to these systems is limited, impractical, and becomes cumbersome to use when exercising in a recreational setting. This has motivated the development of alternative and more practical approaches to traditional BFR resistance exercise that may enhance accessibility and subsequent use in the general population (Loenneke et al., 2012a; Wilson et al., 2013). This alternative approach has been termed practical BFR resistance training and consists of utilizing elastic bands wrapped around the working limbs to reduce blood flow, rather than the standard pneumatic cuffs. While preliminary results have indicated the potential efficacy of this practical approach (Wilson et al., 2013), to our knowledge, only one study (Thiebaud et al., 2019) has directly compared the acute responses between traditional and practical BFR resistance exercise in the same study. Therefore, the ability to prescribe or recommend the use of more practical approaches requires further investigation.

Additionally, little is known regarding any potential physiological differences between males and females to BFR resistance exercise. Most studies have not stratified men and women for analysis or simply have omitted women completely. In fact, female populations are often underrepresented in terms of scientific evaluation, largely attributed to the dynamic hormonal fluctuations of the menstrual cycle or differences between hormonal contraceptive use (Miller, 2014; Hunter, 2016), which may alter their responses or increase variability to exercises used in the studies. Interestingly, Counts et al. (2018) highlighted the exclusion of female participants in BFR research, and specifically noting that the few studies that have included female participants grouped them with men and ignored potential sex differences. Undoubtedly, the influence of sex regarding resistance exercise combined with BFR has not received much attention, highlighting a gap in the present literature. Hence, in order to further develop and optimize BFR resistance training protocols, understanding potential differences in physiological responses between males and females warrants investigation.

Therefore, the purpose of the present study was twofold: (1) examine the acute physiological responses of traditional and practical BFR resistance exercise compared to those from conventional high- and low-load resistance exercise without BFR; and (2) investigate if males and females display different physiological responses to resistance exercise within each exercise condition. It was hypothesized that similar physiological responses would be observed between the traditional and practical BFR resistance exercise protocols. It was also hypothesized that high-load resistance exercise would induce the largest acute responses. Finally, we hypothesized that males would display greater physiological responses than females within all trials.

## Materials and Methods

### Participants

Twenty-nine recreationally active individuals (males: *n* = 14, females: *n* = 15) who had not been engaged in any resistance training program for the previous 6 months volunteered for the study. All participants were normotensive, free from any musculoskeletal injuries and cardiovascular diseases, had a body mass index <30 kg/m^2^, and an ankle brachial index between 0.9 and 1.4. All female participants were actively using hormonal contraceptives and had been using the same contraceptive for the prior 6 months. This research was approved by the University’s Institutional Review Board (IRB no. 8715) and each participant provided informed consent prior to study enrollment.

### Study Design

This study was a randomized, cross-over design that investigated the acute responses in myoelectric activity [surface electromyography (sEMG) amplitude], whole-blood lactate (WBL), muscle swelling (muscle thickness and thigh circumference), hematocrit levels, and plasma volume changes, before and after the following randomized exercise conditions: (1) low-load resistance exercise with traditional BFR (tBFR: 30% of 1-RM and 50% of total arterial occlusion pressure), (2) low-load resistance exercise with practical BFR (pBFR: 30% of 1-RM and 7 on a perceived pressure scale), (3) low-load resistance exercise without BFR (LL: 30% of 1-RM), and (4) high-load resistance exercise without BFR (HL: 80% of 1-RM). Participants attended the laboratory for a total of 6 visits. During the first visit, each participant was provided with an explanation of the study procedures and filled out all forms and questionnaires, followed by measurements of standing height, body weight, brachial blood pressure, ankle brachial index, and completion of a familiarization session with the 1-RM tests. During the second visit, participants’ total arterial occlusion pressure for the lower-body was determined and participants completed the 1-RM tests, in addition to completing a familiarization session with each exercise conditions (tBFR, pBFR, HL, and LL). During the last four visits (visits 3 to 6), participants randomly completed each one of the four experimental conditions. There was a minimal and maximum washout period of 3 and 7 days between trials, respectively, and participants were not tested if they were sore from the previous visit.

### Determination of the Restrictive Pressures

The restrictive pressure to be used during the tBFR trial was determined individually for each participant and based on the total arterial occlusion pressure for the lower body, measured in the posterior tibial artery. After resting for approximately 10 min in the supine position, the participants’ brachial blood pressure was measured using a portable automatic monitor (BP710, OMRON, Chicago, IL, United States). Then, a 13.5 cm wide nylon cuff (SC12, D.E. Hokanson, Bellevue, WA, United States) connected to a rapid inflator system (E20 Rapid Cuff Inflator, D. E. Hokanson, Bellevue, WA, United States), was placed at the most proximal portion of the thigh and inflated to 50 mmHg for 30 s. At the same time, a handheld bidirectional Doppler probe (MD6 Doppler, D. E. Hokanson, Bellevue, WA, United States) coated with transmission gel was positioned over the posterior tibial artery to detect the auscultatory pulse. Next, the cuff was inflated to a pressure equivalent to the person’s systolic blood pressure for about 10 s and then deflated. From there, repeated cycles of inflation and deflation were completed with pressure increments of 10 mmHg per cycle, until the auscultatory pulse could no longer be captured by the Doppler. When that happened, the pressure was slowly and progressively decreased until the pulse could be re-detected. The pressure immediately before re-detection of the pulse was considered the total arterial occlusion pressure. This procedure was repeated in the contralateral limb and the values from both legs were averaged and considered the total arterial occlusion pressure for the lower body. For the pBFR trial, an attempt to individualize the restrictive pressure was performed by using the perceived pressure scale of 0 to 10 (Wilson et al., 2013), in which the investigator would wrap an elastic wrap at the most proximal portion of the leg and tighten it until the participant stated that a perceived pressure of 7 had been reached.

### Maximum Dynamic Muscular Strength Test

Participants performed a 1-RM test for the bilateral horizontal leg press and knee extension exercises (Cybex International Inc., Medway, MA, United States), which served as a parameter to determine the load lifted in each exercise trial. Before starting the test, participants were introduced to proper technique and performed an initial warmup with a load that easily allowed the completion of 8 to 10 repetitions; then, the weight was increased, and participants completed 4 to 5 repetitions; next, the weight was increased again, and participants performed 2 to 3 repetitions. Following the warmups, the weight was progressively increased until the participant was no longer able to lift the load. Participants were given 2–4 min to rest between warmups and between each maximal attempt. The 1-RM was considered the last load lifted with proper form through a full range of motion. The 1-RM for each participant was found within 3 to 5 attempts. There was a minimum rest period of 3 min rest period between the 1-RM test for the leg press and the knee extension exercises.

### Surface Electromyography

Surface electromyography was used to assess the myoelectrical activity of the vastus lateralis muscle of the dominant leg. Bipolar electrodes (EL503, Biopac System, Inc., Goleta, CA, United States) were placed over the belly of the muscle with a 20 mm distance between electrodes at 2/3 of the distance between the anterior spina iliac superior to the lateral side of the patella, following the SENIAM’s recommendations. A semi-permanent ink was used to mark the sites for initial electrode placement in an attempt to ensure that electrodes were placed at the same locations during each visit. The electrode was connected to an amplifier and digitizer system (MP 100, Biopac System, Inc., Goleta, CA, United States), while a ground electrode was placed at the top of the patella. The signal was captured at a frequency of 2000 Hz and stored in a portable computer for analysis using the AcqKnowledge software (AcqKnowledge 3.8.1, Biopac System, Inc., Goleta, CA, United States). Before analysis, the signal was filtered using a low- and a high-pass filter of 500 and 10 Hz, respectively. Normalization of the EMG signal was performed using the signal obtained during a maximum voluntary dynamic contraction (MVDC) for the leg press and knee extension exercises after a warmup (10 repetitions at 50% 1-RM), performed immediately before each one of the four experimental testing visits. The load used during the MVDC corresponded to the participants’ 1-RM load. The concentric portion of each contraction was isolated from the eccentric portion using the event markers function available on the AcqKnowledge software to allow separate analysis. Root mean squares were calculated for the largest 0.5 s interval within the concentric portion of the first and last three repetitions of each set, which were averaged and used to determine the mean sEMG amplitude for each set.

### Whole-Blood Lactate

Whole-blood lactate was measured using a portable lactate analyzer (Lactate Plus, Nova Biomedical Corporation, Waltham, MA, United States) at baseline, immediately post-, 5 min post-, and 15 min post-exercise. Blood samples of approximately 5 μl were collected through finger pricks performed in the index or middle fingers. Before collecting the blood, the finger was wiped with alcohol and the first droplet was discarded. The lactate analyzer was calibrated every day before data collection using low and high lactate standards (Lactate Plus, Nova Biomedical Corporation, Waltham, MA, United States), following the manufacture’s recommendations. The day-to-day reliability for baseline WBL levels was ICC = 0.494. WBL values were corrected for changes in plasma volume and used for statistical analysis.

### Muscle Swelling

Muscle swelling was estimated using muscle thickness and thigh circumference measurements performed at the 50% site of the femur (the halfway point between the lateral condyle of the femur and the great trochanter) of the dominant leg at baseline, immediately post-, 5 min post-, and 15 min post-exercise. Muscle thickness was assessed using an ultrasound device (FF Sonic UF-4500, Fukuda Denshi, Tokyo, Japan) and a 5-MHz scanning head coated with transmission gel. Muscle thickness consisted of the perpendicular distance from the adipose tissue-muscle interface to the muscle-bone interface. Thigh circumference was measured using a tape measure wrapped around the thigh at the same 50% site, following each muscle thickness measurement. During both muscle thickness and ultrasound measurements, participants were instructed to stand still, with legs positioned shoulder width apart, and distribute their body weight equally between both legs. Each measurement was performed by the same trained technician to the nearest tenth of a centimeter. The day-to-day reliability for baseline muscle thickness and circumference values were ICC = 0.981 and ICC = 0.983, respectively.

### Hematocrit Levels and Plasma Volume Changes

Finger pricks were also used to determine hematocrit levels (Hct) and percent changes in plasma volume (%ΔPV) at baseline, immediately post-, 5 min post-, and 15 min post-exercise. Whole blood was collected into a heparinized plastic micro-hematocrit tube and centrifuged. Blood samples for each time point were collected in duplicate and averaged to determine the respective Hct (%) and %ΔPV values. Hct (%) was considered the percent of whole blood that is red blood cells, and it was determined using a micro-capillary reader (Damon/IEC Division, Needham, MA, United States) in each sample. %ΔPV were determined by the following equation proposed by Van Beaumont (1972):$\%\Delta \mathrm{PV}=\left(\frac{100}{100-{\mathrm{Hct}}_{\text{pre}}}\right)\times 100\times \left(\left(\frac{{\text{Hct}}_{\text{pre}}}{{\text{Hct}}_{\text{pre}}-{\text{Hct}}_{\text{post}}}\right)/{\text{Hct}}_{\text{post}}\right)$. The day-to-day reliability for baseline hematocrit levels was ICC = 0.909.

### Exercise Protocols

Participants randomly performed the following experimental conditions: (1) tBFR, consisting of four sets of 30-15-15-15 repetitions of the bilateral horizontal leg press and knee extension exercises, at 30% of 1-RM, with a 13.5 cm wide pneumatic cuff placed at the most proximal portion of each thigh and inflated to a pressure equivalent to 50% of the participant’s total occlusion pressure; (2) pBFR, which was the same as the tBFR condition, except that an elastic band (5 cm wide) wrapped around the proximal portion of each thigh at a tightness of 7 on a 0 to 10 perceived pressure scale (Wilson et al., 2013) was used to reduce blood flow; (3) LL, which was similar to the tBFR and pBFR protocols, but no BFR was applied; and (4) HL, consisting of three sets of 10 repetitions at 80% of 1-RM, with the same 1-min rest interval between sets and a 3-min rest period between exercises. A digital metronome set at 40 beats per minute was used to guarantee a contraction speed of 1.5 s for the concentric and eccentric portions of the contraction for all exercise protocols. Prior to each exercise condition, a warmup consisting of 8 to 10 repetitions at 30% of the participant’s 1-RM were performed for both exercises. All immediately post-exercise measurements were taken following cuff deflation or removal of the elastic bands for the tBFR and pBFR protocols, respectively.

### Statistical Analyses

All analyses were performed in R studio 3.6.1 (R Foundation for Statistical Computing, Vienna, Austria). Data normality was confirmed before any statistical analyses were performed using the Shapiro–Wilk test and graphical information from histograms and Q-Q plots. Homogeneity of variance was confirmed using the Fisher’s test, and then descriptive statistics were compared between males and females using independent sample *t*-tests, followed by the calculation of Cohen’s *d* as mean change divided by the pooled standard deviation of the change (for sex comparisons) or average standard deviation (for the comparisons across conditions) as an estimate of effect size, which were interpreted as suggested by Rhea (2004). A mixed model 3-way repeated measures analysis of variance (ANOVA) [sex × condition × time] was used to test for significant main effects and interactions. In the case of significant interactions, simple effects were tested using separate pairwise *t*-tests with the Bonferroni procedure to control for the familywise error rate. Greenhouse–Geisser correction was used in the case of non-sphericity and generalized eta-squares ($\eta_{\text{G}}^{2}$) were calculated as estimates of effect size, and interpreted as follows: 0.02 as small, 0.13 as medium, and 0.26 as a large effect size (Cohen, 1988). Main effects were interpreted only if interactions were absent. Statistical analyses for sEMG amplitude during exercises was divided into two separate analyses. The initial analysis included only the first three sets of each exercise condition, since the HL condition did not include a fourth set. The second analysis consisted of the remaining fourth sets of the three low-load resistance exercise conditions not included in the first analysis. Intraclass correlation coefficient estimates were calculated using the baseline values of WBL, muscle thickness, and thigh circumference, based on an absolute agreement, two-way mixed-effects model. An *a priori* sample size calculation using G^∗^Power 3.1 (Franz Faul, University of Kiel, Germany) determined that 30 participants would be required to detect an effect size of at least 0.3 (α = 0.05, β = 0.80, number of groups = 6, number of measurements = 4, correlation among variables = 0.06). Data are presented as means ± standard deviations, and the level of significance was set at α ≤ 0.05.

## Results

### Participants’ Characteristics

The oral contraceptives’ common and generic names as well as their respective dosages are outlined in Table 1, and participants’ characteristics are presented in Table 2. Males displayed significantly (*p* < 0.001) greater mean values for age, body weight, standing height, and maximum strength levels for the leg press and knee extension exercises, whereas no significant (*p* > 0.05) differences were observed between sexes for BMI or averaged BFR occlusion pressure for both legs. Participants were able to complete the pre-determine number of repetitions for the three low-load exercise conditions but not for the HL trial (29.5 ± 2.4) during leg press, while the average number of repetitions completed for the tBFR, pBFR, LL, and HL trials during knee extension was 72.5 ± 5.1, 74.6 ± 1.5, 22.1 ± 4.6, and 74.2 ± 2.6, respectively.

**TABLE 1:** Hormonal contraceptives used and their respective dosages.

**Common name**	**Generic name and dosage**
Nikki	Ethinyl Estradiol 0.02 mg, Drospirenone 3 mg
Estrostep Fe	Norethindrone acetate 0.5 mg, ethinyl estradiol 2.5 mcg
Skyla	Levonorgestrel-releasing intrauterine system 13.5 mg
Lo Loestrin Fe	Norethindrone acetate 1 mg, ethinyl estradiol 0.01 mg; ethinyl estradiol 0.01 mg
Blisovi Fe	Ethinyl estradiol 0.02 mg, norethindrone acetate 1 mg
NORG-EE	Norgestimate, Ethinyl Estradiol (0.18–0.215–0.25–0.035 mg)
Depo-provera	Medroxyprogesterone acetate 150 mg/mL
Loestrin	Norethindrone-ethin estradiol 1 mg/0.02 mg
Daysee	Ethinyl estradiol 0.03 mg, levonorgestrel 0.15 mg, ethinyl estradiol 0.01 mg
Tridione	Trimethadione 150 mg
Junel Fe	Norethindrone acetate 1 mg, ethinyl estradiol 20 mcg, ferrous fumarate 75 mg

**TABLE 2:** Participants’ characteristics.

	**Total (*n* = 29)**	**Males (*n* = 14)**	**Females (*n* = 15)**	***t***	***p*-value**	***d* (95% CI)**
Age (years)	21.90 ± 2.70	23.57 ± 2.65*	20.33 ± 1.63	3.99	< 0.001	2.49 (1.50, 3.46)
Weight (kg)	71.97 ± 12.30	80.86 ± 10.07*	63.67 ± 7.45	5.24	< 0.001	1.95 (1.05, 2.83)
Height (m)	1.71 ± 0.10	1.78 ± 0.06*	1.65 ± 0.07	5.66	< 0.001	2.10 (1.17, 3.01)
BMI (kg/m^2^)	24.34 ± 2.73	25.34 ± 3.11	23.41 ± 1.99	2.01	0.055	0.75 (−0.01, 1.49)
Total occlusion pressure (mmHg)	139.16 ± 4.30	143.60 ± 13.09	135.00 ± 14.55	1.67	0.106	0.62 (−0.13, 1.36)
Leg Press 1-RM (kg)	144.43 ± 39.65	173.51 ± 32.32*	117.29 ± 23.22	5.41	< 0.001	2.01 (1.09, 2.90)
Knee Extension 1-RM (kg)	85.15 ± 27.93	107.61 ± 21.29*	64.19 ± 12.80	6.71	< 0.001	2.49 (1.50, 3.46)

*Significantly greater than females (*p* < 0.01), *d* = Cohen’s *d* effect size. Data are mean ± SD.

### Surface Electromyography

#### Leg Press

**FIGURE 1:**
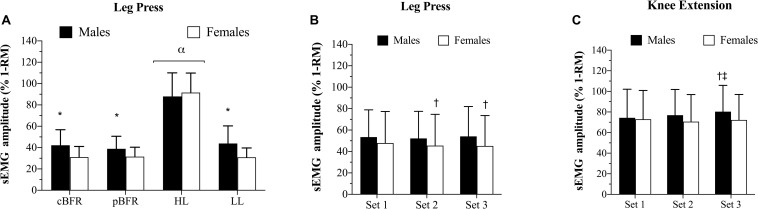
**(A)** Surface electromyography sex × condition interaction for leg press, **(B)** sex × set interaction for leg press, **(C)** sex × set interaction for knee extension. tBFR: traditional blood flow restriction resistance exercise condition, pBFR: practical blood flow restriction resistance exercise condition, HL: high-load resistance exercise condition, LL: low-load resistance exercise condition. *Significantly greater than females (*p* < 0.05), ^α^ Significantly greater than all conditions (*p* < 0.05), ^†^Significantly different than set 1 (*p* < 0.05), ^‡^Significantly different than set 2 (*p* < 0.05).

Table 3 outlines the mean sEMG amplitude values for each set completed during all experimental trials for both leg press and knee extension exercises. There were significant sex × condition (*p* = 0.018, *F* = 3.57, $\eta_{\text{G}}^{2}$ = 0.05) and sex × set (*p* = 0.03, *F* = 3.74, $\eta_{\text{G}}^{2}$ < 0.01) interactions for sEMG amplitude during the first 3 sets of leg press for all exercise conditions. As demonstrated in Figure 1A, males displayed significantly (*p* < 0.01, *d* = 0.72 to 0.90) greater myoelectrical activity than females within all exercise conditions, except HL (*p* = 0.42), which also elicited significantly (*p* < 0.01) greater myoelectrical activity compared to tBFR, pBFR, and LL for both sexes. Additionally, no significant (*p* > 0.05) differences existed between the tBFR and pBFR trials within males or females. Further analyses of the sex × set interaction (Figure 1B) revealed that males presented similar (*p* > 0.05) sEMG amplitude compared to females during all sets, and that no significant (*p* > 0.05) differences existed across sets for males, whereas set 1 was significantly (*p* < 0.01) greater than sets 2 and 3 for females. In regard to the fourth set of exercise for the three low-load conditions, there was only a significant sex main effect (*p* = 0.004, *F* = 9.79, $\eta_{\text{G}}^{2}$ = 0.19) with males (41.99 ± 15.54 %MVDC) displaying greater sEMG amplitude than females (30.11 ± 8.62 %MVDC).

**TABLE 3:** Surface electromyography amplitude (%MVDC) per set during all experimental trials for males and females.

**Leg Press**					
		**Set 1**	**Set 2**	**Set 3**	**Set 4**
Males	tBFR	41.95 ± 13.70	42.06 ± 14.88	42.59 ± 16.06	44.08 ± 17.23^‡^
	pBFR	39.38 ± 11.19	37.98 ± 11.78	39.27 ± 12.94	38.04 ± 12.63
	HL	87.18 ± 21.58	85.69 ± 20.24	91.14 ± 25.29	−
	LL	45.11 ± 17.64	43.06 ± 17.56	43.52 ± 15.35	43.88 ± 17.12
Females	tBFR	33.18 ± 9.14	31.12 ± 10.63	28.78 ± 10.36	28.90 ± 10.0
	pBFR	33.04 ± 8.54	30.21 ± 8.87	30.96 ± 9.58	30.95 ± 8.50
	HL	93.89 ± 19.58	90.90 ± 19.84	89.66 ± 16.70	−
	LL	31.36 ± 9.79	29.71 ± 8.15	31.25 ± 8.88	30.47 ± 7.67

**Knee Extension**					
		**Set 1**	**Set 2**	**Set 3**	**Set 4**

Males	tBFR	65.34 ± 21.31	72.23 ± 23.49	75.49 ± 24.93	78.77 ± 24.04
	pBFR	62.67 ± 18.89	68.97 ± 22.66	71.19 ± 20.57	76.28 ± 21.97
	HL	107.10 ± 27.18^α^	100.50 ± 21.75^α^	104.76 ± 24.70^α^	−
	LL	62.29 ± 12.93	65.97 ± 14.87	69.79 ± 14.87*	72.89 ± 17.68
Females	tBFR	68.93 ± 20.90	63.31 ± 13.00	70.22 ± 15.73^βγ^	75.26 ± 16.95
	pBFR	60.48 ± 15.46	58.23 ± 14.63	61.99 ± 16.62	65.01 ± 18.22
	HL	104.25 ± 30.45^α^	104.49 ± 27.03^α^	102.04 ± 23.22^α^	−
	LL	57.84 ± 14.91	56.48 ± 11.37	54.93 ± 10.91	59.05 ± 10.93

tBFR: traditional blood flow restriction resistance exercise condition, pBFR: practical blood flow restriction resistance exercise condition, HL: high-load resistance exercise condition, LL: low-load resistance exercise condition. *Significantly different than females within the same set (*p* ≤ 0.01). ^α^Significantly greater than tBFR, pBFR, and LL within the same sex (*p* < 0.05). ^β^Significantly greater than pBFR within the same sex (*p* < 0.05). ^γ^Significantly different than LL within the same sex (*p* < 0.05).

#### Knee Extension

For the knee extension exercise, there were significant sex × set (*F* = 3.54, *p* = 0.035, $\eta_{\text{G}}^{2}$ < 0.01) and sex × condition × set (*p* = 0.039, *F* = 2.67, $\eta_{\text{G}}^{2}$ < 0.01) interactions for the first three sets of all four experimental conditions. Follow-up analyses of the sex × set interaction (Figure 1C) demonstrated that no significant (*p* > 0.05) differences existed between males and females from sets 1 to set 3 (Figure 1C), and that set 3 was significantly (*p* < 0.01) greater than sets 1 and 2 for males, while no significant (*p* > 0.05) differences across sets existed for females. Further breakdown of the three-way interaction (Table 2) demonstrated that there were no significant sex differences (*p* > 0.05) from set 1 to set 3 within each experimental condition, except for the LL condition during set 3 set, in which males were significantly (*p* < 0.01, *d* = 1.15) greater than females. For the comparisons across conditions within sexes and sets, no significant (*p* > 0.05) differences existed between tBFR, pBFR, and LL from set 1 to set 3, while HL was significantly greater than all conditions during all sets for males; the same results were observed for females, except that tBFR was significantly (*p* < 0.01) greater than pBFR and LL during set 3. Finally, for the analysis including only the fourth set of the three low-load conditions, there was only a significant condition main effect (*p* = 0.005, *F* = 5.79, $\eta_{\text{G}}^{2}$ = 0.06) with tBFR (76.95 ± 20.37 %MVDC) being significantly (*p* < 0.01) greater than LL (65.72 ± 15.95 %MVDC), but similar (*p* = 0.08) to pBFR (70.46 ± 20.57 %MVDC).

### Whole-Blood Lactate

**FIGURE 2:**
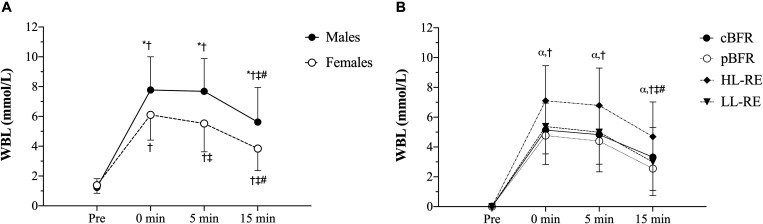
**(A)** Whole-blood lactate (WBL) sex × time interaction, **(B)** WBL condition × time interaction. tBFR: traditional blood flow restriction resistance exercise condition, pBFR: practical blood flow restriction resistance exercise condition, HL: high-load resistance exercise condition, LL: low-load resistance exercise condition. *Significant sex difference (*p* < 0.05), ^α^HL significantly greater than tBFR, pBFR, and LL (*p* < 0.05), ^†^Significantly different than pre (*p* < 0.05), ^‡^Significantly different than 0 min (*p* < 0.05), ^#^Significantly different than 5 min (*p* < 0.05). Data are mean ± SD absolute change from baseline.

Table 4 outlines the time course changes in WBL for males and females following each experimental condition. There were significant sex × time (*p* = 0.001, *F* = 9.93, $\eta_{\text{G}}^{2}$ = 0.07) and condition × time interactions (*p* < 0.001, *F* = 16.02, $\eta_{\text{G}}^{2}$ = 0.06) for WBL. Further analyses revealed that males displayed significantly (*p* < 0.01) greater WBL levels than females immediately post (*d* = 0.92), 5 min (*d* = 1.13), and 15 min post-exercise (*d* = 1.00), and that WBL peaked at 5 min post-exercise for males and immediately post-exercise for females (Figure 2A). Regarding the condition × time interaction (Figure 2B), pairwise comparisons demonstrated that no significant (*p* > 0.05) differences existed between the tBFR and pBFR trials, except at 15 min post-exercise when tBFR was significantly (*p* = 0.04, *d* = 0.4) greater than pBFR, while the HL condition elicited the greatest (*p* < 0.05) increase in WBL in comparison to the tBFR (*d* = 0.69 to 0.95), pBFR (*d* = 1.04 to 1.13), and LL (*d* = 0.77 to 0.87) protocols at all post-exercise time points. Finally, immediately post-exercise and 5 min post-exercise WBL levels were significantly greater than pre-exercise and 15 min post-exercise values for all testing conditions.

**TABLE 4:** Time course changes in whole blood lactate (mmol/L) for males and females within each experimental condition.

		**Pre-exercise**	**Immediately post**	**5 min post**	**15 min post**
Males	tBFR	1.34 ± 0.63	7.42 ± 1.91	7.16 ± 2.82	5.46 ± 1.32
	pBFR	1.21 ± 0.69	7.92 ± 2.62	7.86 ± 2.58	4.78 ± 1.50
	HL	1.11 ± 0.49	9.30 ± 2.53	9.22 ± 3.49	7.96 ± 2.52
	LL	1.29 ± 0.52	7.43 ± 2.19	7.49 ± 2.21	5.59 ± 2.46
Females	tBFR	1.45 ± 0.50	5.70 ± 1.39	5.36 ± 1.64	4.32 ± 1.31
	pBFR	1.52 ± 0.74	5.40 ± 1.80	4.75 ± 2.93	3.12 ± 1.33
	HL	1.24 ± 0.39	7.33 ± 1.58	7.79 ± 1.78	4.85 ± 1.46
	LL	1.33 ± 0.50	5.99 ± 1.46	5.21 ± 1.75	3.38 ± 1.33

*tBFR: traditional blood flow restriction resistance exercise condition, pBFR: practical blood flow restriction resistance exercise condition, HL: high-load resistance exercise condition, LL: low-load resistance exercise condition. Data are raw mean ± SD.*

### Muscle Swelling

**FIGURE 3:**
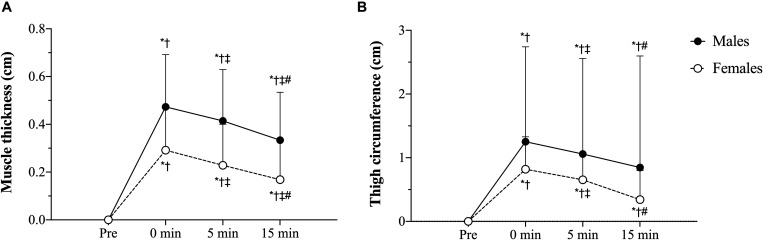
**(A)** Muscle thickness sex × time interaction, **(B)** Thigh circumference sex × time interaction. tBFR: traditional blood flow restriction resistance exercise condition, pBFR: practical blood flow restriction resistance exercise condition, HL: high-load resistance exercise condition, LL: low-load resistance exercise condition. ^∗^Significant sex difference (*p* < 0.05), ^†^Significantly different than pre (*p* < 0.05), ^‡^Significantly different than 0 min (*p* < 0.05), ^#^Significantly different than 5 min (*p* < 0.05). Data are mean ± SD absolute change from baseline.

Table 5 outlines the time course changes in muscle thickness and thigh circumference for males and females following each experimental condition. There was a significant sex × time interaction (*p* = 0.001, *F* = 9.59, $\eta_{\text{G}}^{2}$ < 0.01) with pairwise comparisons revealing significant (*p* < 0.01) sex differences in which males displayed greater muscle thickness than females at baseline (*d* = 1.24), immediately post- (*d* = 0.99), 5 min post- (*d* = 0.96), and 15 min (*d* = 0.92) post-exercise (Figure 3A). Additionally, the increases in muscle thickness peaked immediately post-exercise (*p* < 0.01) and remained elevated from baseline levels up to 15 min post for (*p* < 0.01) males and females.

**TABLE 5:** Absolute muscle thickness (cm) and thigh circumference (cm) values for males and females following each experimental condition.

**Muscle thickness**					
		**Pre-exercise**	**Immediately post**	**5 min post**	**15 min post**
Males	tBFR	5.85 ± 0.81	6.34 ± 0.83	5.81 ± 0.79	6.18 ± 0.83
	pBFR	5.81 ± 0.79	6.29 ± 0.82	6.21 ± 0.82	6.14 ± 0.83
	HL	5.82 ± 0.73	6.28 ± 0.81	6.26 ± 0.77	6.20 ± 0.78
	LL	5.78 ± 0.81	6.25 ± 0.92	6.17 ± 0.89	6.07 ± 0.89
Females	tBFR	4.90 ± 0.65	4.89 ± 0.68	5.11 ± 0.67	5.07 ± 0.67
	pBFR	4.89 ± 0.67	5.17 ± 0.66	5.12 ± 0.65	5.04 ± 0.65
	HL	5.01 ± 0.81	5.33 ± 0.79	5.26 ± 0.83	5.11 ± 0.64
	LL	4.87 ± 0.64	5.17 ± 0.66	5.11 ± 0.65	5.03 ± 0.62

**Thigh circumference**					
		**Pre-exercise**	**Immediately post**	**5 min post**	**15 min post**

Males	tBFR	55.86 ± 5.49	57.82 ± 4.39	57.62 ± 4.36	57.77 ± 4.47
	pBFR	56.62 ± 4.31	57.64 ± 4.33	57.50 ± 4.33	56.94 ± 4.67
	HL	56.93 ± 4.34	58.01 ± 4.46	57.74 ± 4.35	57.54 ± 4.28
	LL	56.41 ± 4.34	57.37 ± 4.41	57.21 ± 4.35	56.97 ± 4.38
Females	tBFR	54.03 ± 4.40	54.90 ± 4.47	54.69 ± 4.33	54.38 ± 4.31
	pBFR	54.21 ± 4.67	54.90 ± 4.74	54.90 ± 4.74	54.57 ± 3.92
	HL	54.16 ± 3.92	54.84 ± 4.08	54.84 ± 4.08	54.55 ± 4.02
	LL	54.13 ± 4.07	56.97 ± 4.38	54.71 ± 4.14	54.39 ± 4.23

*tBFR: traditional blood flow restriction resistance exercise condition, pBFR: practical blood flow restriction resistance exercise condition, HL: high-load resistance exercise condition, LL: low-load resistance exercise condition. Data are raw mean ± SD.*

Regarding thigh circumference, there was also a significant sex × time interaction (*p* = 0.046, *F* = 3.56, $\eta_{\text{G}}^{2}$ < 0.01). Follow-up analyses demonstrated that, similar to muscle thickness, males presented significantly (*p* ≤ 0.05) greater thigh circumference values than females at baseline (*d* = 0.49), immediately post- (*d* = 0.39), 5 min post- (*d* = 0.37), and 15 min (*d* = 0.40) post-exercise (Figure 3B) Lastly, the increases in muscle thickness peaked immediately post-exercise (*p* < 0.01) and remained elevated from baseline levels up to 15 min post for (*p* < 0.01) both sexes.

### Hematocrit (Hct) and Plasma Volume (PV)

For hematocrit values (Table 6), there was only a significant condition × time interaction (*p* = 0.005, *F* = 3.15, $\eta_{\text{G}}^{2}$ = 0.01), with pairwise comparisons revealing that the changes in hematocrit for HL, tBFR, and pBFR were significantly (*p* < 0.01) greater than LL immediately post-exercise; HL (*p* < 0.01) and tBFR (*p* = 0.04) were significantly greater than LL 5 min post; whereas HL and tBFR were significantly (*p* ≤ 0.01) greater than LL 15 min post.

**TABLE 6:** Time course changes in hematocrit and plasma volume for males and females following each experimental condition.

**Hematocrit**					
		**Pre-exercise**	**Immediately post**	**5 min post**	**15 min post**
Males	tBFR	46.18 ± 2.83	47.68 ± 2.13	47.93 ± 2.50	45.75 ± 2.35
	pBFR	45.32 ± 2.09	46.64 ± 2.08	46.50 ± 2.70	45.29 ± 2.01
	HL	45.82 ± 2.28	47.29 ± 2.38	47.46 ± 2.73	45.89 ± 2.03
	LL	46.32 ± 3.38	46.86 ± 3.09	46.39 ± 3.50	45.07 ± 2.79
Females	tBFR	42.73 ± 2.19	43.46 ± 2.80	43.43 ± 2.09	41.63 ± 2.75
	pBFR	41.60 ± 2.44	42.36 ± 1.90	42.20 ± 2.46	41.53 ± 2.15
	HL	42.07 ± 2.78	43.13 ± 2.75	43.56 ± 2.84	41.83 ± 2.86
	LL	42.57 ± 3.83	42.00 ± 3.22	42.60 ± 3.19	41.00 ± 2.91

**Plasma volume changes**					
		**Pre-exercise**	**Immediately post**	**5 min post**	**15 min post**

Males	tBFR	−	−5.53 ± 8.51	−6.46 ± 8.39	2.08 ± 8.76
	pBFR	−	−5.00 ± 6.08	−4.08 ± 10.63	0.40 ± 7.43
	HL	−	−5.54 ± 6.12	−6.13 ± 7.18	0.04 ± 8.31
	LL	−	−1.98 ± 5.86	−0.06 ± 6.83	5.34 ± 6.28
Females	tBFR	−	−2.75 ± 5.82	−2.65 ± 6.13	4.97 ± 8.46
	pBFR	−	−2.93 ± 6.23	−2.09 ± 8.35	0.57 ± 8.66
	HL	−	−4.14 ± 5.65	−4.76 ± 6.28	1.13 ± 6.13
	LL	−	2.48 ± 5.96	−0.06 ± 5.15	6.83 ± 6.87

*tBFR: traditional blood flow restriction resistance exercise condition, pBFR: practical blood flow restriction resistance exercise condition, HL: high-load resistance exercise condition, LL: low-load resistance exercise condition. Data are raw mean ± SD.*

There were no significant (*p* > 0.05) interactions for changes in plasma volume (Table 5), but there were significant condition (*p* < 0.001, *F* = 6.24, $\eta_{\text{G}}^{2}$ = 0.08) and time (*p* < 0.001, *F* = 59.04, $\eta_{\text{G}}^{2}$ = 0.14) main effects. *Post hoc* analyses revealed that no significant (*p* > 0.05) differences existed between the tBFR, pBFR, and HL protocols, which were all significantly (*p* < 0.05) greater than the LL condition. Further analyses also demonstrated that immediately post- and 5 min post- were significantly greater that 15 min post-exercise measures.

## Discussion

The purpose of this investigation was to provide novel insight comparing the acute physiological responses between tBFR and pBFR to traditional bouts of low- and high-load resistance exercise, while comparing the influence of sex on these responses. The results from this study partially confirm our hypothesis that males would display greater physiological responses compared to females. Furthermore, as hypothesized, tBFR and pBFR elicited similar responses for the physiological parameters tested, however, our results also indicate that HL elicited greater physiological responses compared to both tBFR and pBFR protocols.

For sEMG amplitude, sex differences were observed only during leg press for the three low-intensity protocols in the comparisons within conditions, whereas no differences existed within any of the experimental trials during knee extension. Considering that leg press was always performed prior to knee extension and that both exercises possibly result in different patterns of recruitment, these results may be interpreted in one of two ways: first, the influence of sex on myoelectrical activity disappears as the exercise bout progresses or, second, such influence may be exercise dependent and vary from one exercise to another. Additionally, it has been demonstrated that men display greater percent of type II muscle fiber area than women in the vastus lateralis muscle, the same used for myoelectrical activity measurement in the current study (Staron et al., 2000). Thus, a potential contribution of fiber type difference to the observed sex difference cannot be completely ruled out. Lastly, similar sEMG results were observed for the two BFR exercise conditions, although these were lower than those observed during HL. These results are supported by previous data from our research group that has also demonstrated that HL elicits greater myoelectrical activity compared to tBFR, utilizing the same exercises and muscle groups in a cohort of young adult males (Freitas et al., 2020).

Regarding the metabolic response to the exercise bouts expressed as WBL, our data suggest that males display greater metabolic response compared to females, regardless of the method of exercise performed. This is not surprising considering that males generally possess greater muscle mass (Jaworowski et al., 2002) and, although muscle glycogen stores seem to be similar across sexes (Tarnopolsky et al., 2001; Wismann and Willoughby, 2006), men usually present greater glycolytic enzyme activity (Simoneau et al., 1985; Jaworowski et al., 2002), which in turn results in a larger metabolic response to exercise (i.e., accumulation of hydrogen ions, lactate, inorganic phosphate, etc.). Additionally, as observed in sEMG amplitude, HL also elicited a greater metabolic response compared to all low-load conditions, which may indicate that changes in myoelectrical activity and in the metabolic responses to resistance exercise are primarily driven by the exercise load. Nevertheless, the influence of the exercise-induced metabolic stress on muscle activity in this experiment is not clear, considering that the observed sex difference in the metabolic response to exercise was not reflected in a paralleling sex difference in the measured myoelectrical activity. Such discrepancy may be related to the issues inherent to the sEMG technique itself (Vigotsky et al., 2018).

Sex differences were also detected for our indices of muscle swelling (muscle thickness and thigh circumference). Moreover, there were no significant differences across conditions for any of the muscle swelling parameters following exercise. These results are surprising as significant differences in the measure of metabolic stress were observed between sexes and conditions during exercise. The exercise-induced metabolic response is known for inducing muscle swelling, as the accumulation of metabolites within the muscle increases the intramuscular osmotic pressure and causes a plasma fluid shift inside the muscle, which seems to have happened in this study as indicated by the ≈5% decreases in plasma volume post-exercise. However, it is important to highlight that changes in plasma volume and hematocrit levels do not guarantee that cell swelling occurred, as fluid shift to the interstitial space rather than the intracellular environment could also have occurred; as well as it does not take into account fluid loss due to sweating (Nielsen, 1974). Therefore, as different exercise conditions resulted in distinct metabolic responses, one would expect this to also result in different levels of muscle swelling across conditions. Our hypothesis explaining this phenomenon is that there is potentially a limit to the extent to which the exercise-induced metabolic stress may contribute to the muscle swelling response post-exercise. Hence, once a certain level of metabolic stress is reached, a further increase in the metabolite accumulation will not necessarily result in a further increase in muscle swelling. This phenomenon has also been observed in previous research with tBFR and HL eliciting similar levels of muscle thickness post-exercise, despite much greater lactate levels measured following HL (Freitas et al., 2020).

Our results are also in agreement with previous literature. Thiebaud et al. (2019) compared the acute changes in muscle thickness and sEMG amplitude in response to traditional low- (30% of 1-RM) and high-load (70% of 1-RM) resistance exercise as well as low- and high-pressure low-load resistance with practical and traditional BFR. Low- and high-pressures for the pBFR condition were determined based off the amount of stretch applied to the elastic wraps (2 in. stretch from resting length or 80% of the person’s thigh circumference), while pressures of 40 and 80% of total BFR were used for the tBFR protocol. Similar to the current investigation, the authors reported no significant differences in muscle thickness changes across any of the tested conditions; however, a greater increase in sEMG was observed for high-pressure pBFR compared to low-pressure tBFR. However, in our view, such difference seems to be more related to the amount of pressure applied than the BFR resistance exercise technique used. Moreover, Thiebaud et al. (2019) had participants perform the exercise to volitional failure while participants completed a pre-determined number of repetitions in the current study. Wilson et al. (2013) also compared pBFR to conventional low-load resistance exercise and reported a greater average post-exercise (i.e., 1, 5, and 10 min) lactate response for the pBFR and greater sEMG amplitude during the set of exercise, and, although muscle thickness did not statistically differ across conditions, significant increases occurred following pBFR exercise while no changes took place as a results of conventional LL resistance exercise. Although no difference were observed between the pBFR and LL conditions in the current study, the overall post-exercise (1 to 15 min) WBL for the pBFR trial for the males subjects in our study is in line with that reported by Wilson et al. (2013) (i.e., 6.19 ± 1.49 versus 6.20 ± 2.80 mmol/L, respectively). The relatively larger WBL levels observed in our study following LL compared to that from Wilson et al. (2013) may be due to the fact that the authors included participants with 1 year of resistance training experience, while only recreationally active individuals partook in our study. Similarly, differences in training status may also underlie the observed differences sEMG amplitude results across both studies. In fact, Loenneke et al. (2010) reported no difference in WBL between pBFR and LL protocols, during or after exercise in recreationally active individuals, although it should be recognized that the authors used intermittent BFR.

It is important to highlight that HL elicited much greater myoelectrical activity and metabolic response compared to both BFR exercise conditions (traditional and practical). The exercise-induced metabolic response and changes in the sEMG amplitude have been referred to as potential contributing factors for the increases in muscular size and strength, commonly observed following BFR resistance training (Pearson and Hussain, 2014). In many cases, the increases in muscular size have been reported to be comparable to those observed with traditional high-load resistance training (Lixandrão et al., 2015, 2018). Thus, we previously hypothesized that the changes in all physiological markers measured would be somewhat similar between the HL and BFR trials, which turned out not to be the case. This called our attention because, if changes in sEMG and WBL do contribute to the positive BFR resistance exercise long-term adaptations, and considering that previous research has observed similar changes in muscle size between HL and BFR resistance exercise, one would also expect the changes in WBL and sEMG amplitude not to differ at a such extent. In fact, Takada et al. (2012) demonstrated the changes in muscle mass occurring after 4 weeks of BFR resistance exercise (≈14%) were highly correlated with the changes in inorganic phosphate concentration and pH decrease. On the other hand, previous studies have reported conflicting results, including no difference between BFR and HL (Suga et al., 2010, 2012), BFR greater than HL (Takarada et al., 2000b), and BFR lower than HL (Suga et al., 2009). It is not clear to us what caused such difference in the exercise-induced metabolic stress and myoelectrical activity observed in this study. Interestingly, Morton et al. (2019) observed similar intramuscular glycogen depletion in types I and II muscle fibers following high-load (80% of 1-RM) and low-load (30% of 1-RM) resistance exercise, regardless of greater sEMG amplitude observed during the high-load condition. These findings suggest that activation of the higher threshold type II muscle fiber may occur during low-load resistance exercise without resulting in a greater sEMG amplitude.

This study includes a few limitations that warrant further discussion. Previous studies have questioned the precision and reliability of the perceived pressure scale used in the current study (Bell et al., 2018, 2020). Additionally, we were unable to determine if the method used to standardize the restrictive pressure of the pBFR trial resulted in the same levels of restriction across participants or if it was equivalent to the 50% restrictive pressure used in the tBFR trial; however, it has been demonstrated that this method induces reduction of arterial BFR and occlusion of venous return (Wilson et al., 2013), which is the ideal scenario for BFR resistance exercise. In addition to that, the occlusion pressure was measured with subjects lying down, while tBFR was performed with subjects in the seated position, which may have interfered with the 50% of BFR applied during exercise. Further, it should be mentioned that the women in the current study had been making use of hormonal contraceptives for the previous 6 months at varying dosages. Therefore, it is plausible that the persistent contraceptive use could influence potential sex differences, thus additional studies investigating sex differences in BFR should include women that are not using hormonal contraceptives to provide further insight on the mechanisms contributing to the observed sex differences among these training modalities.

## Conclusion

In conclusion, this study demonstrated that males and females may display different physiological responses during and following resistance training. Thus, future studies should consider sex as a potential confounding variable. Furthermore, this study also demonstrated that both traditional and practical BFR resistance exercise are capable of inducing the same physiological responses, which provides novel insight into the potential use of pBFR as a more feasible BFR resistance exercise approach to be performed outside of the laboratory environment.

## Data Availability Statement

The datasets generated for this study are available on request to the corresponding author.

## Ethics Statement

The studies involving human participants were reviewed and approved by University of Oklahoma Institutional Review Board. The patients/participants provided their written informed consent to participate in this study.

## Author Contributions

All authors contributed to designing the study, data analyses and interpretation, writing, and proofreading the manuscript, and approved the content of the manuscript’s final version.

## Conflict of Interest

The authors declare that the research was conducted in the absence of any commercial or financial relationships that could be construed as a potential conflict of interest.
